# The reliability of a smartphone goniometer application compared with a traditional goniometer for measuring first metatarsophalangeal joint dorsiflexion

**DOI:** 10.1186/s13047-015-0088-3

**Published:** 2015-07-23

**Authors:** Simon J. Otter, Brunilda Agalliu, Nicola Baer, Georgie Hales, Katrina Harvey, Keeley James, Richard Keating, Warren McConnell, Rachel Nelson, Saddaf Qureshi, Steven Ryan, Abigail St. John, Heather Waddington, Katie Warren, Duane Wong

**Affiliations:** School of Health Science, 49 Darley Rd, Eastbourne, BN20 7UR UK

**Keywords:** Biomechanics Cell phone, Reproducibility of results, First metatarsophalangeal joint

## Abstract

**Background:**

Adequate sagittal plane motion of the first metatarsalphalangeal joint (1st MTPJ) is important during normal gait and goniometric measurement is commonly used as a diagnostic and outcome assessment tool. We aimed to determine the intra and inter-rater reliability together with the concurrent validity of a universal plastic goniometer (UG) and a smartphone applicationlication (Dr G) for the measurement of dorsiflexion at the 1st MTPJ.

**Methods:**

Measurement of joint position and passive range of motion of the 1st MTPJ dorsiflexion was compared using a UG and DrG goniometer. A double-blind repeated measures design was utilized, with intraclass correlation coefficient (ICC) used to determine levels of reliability.

**Results:**

For *joint position* good intra-rater reliability (ICC >0.861) and good inter-rater reliability (ICC >0.823) was noted. However, the Dr G application consistently measured lower angles (mean 27.8° (SD 8.37)) than the UG (mean 32° (SD 11.7)) and these associations were significant (r = 0.399, *p* < 0.001). For *passive range of motion*, the mean total range of dorsiflexion motion (from maximum plantarflexed position to maximum dorsiflexed position) was 82.8° (SD 12.2) for the UG and 82.9° (SD 11.3) for the Dr G application. Both instruments demonstrated high levels of intra-rater reliability (ICC >0.809). Inter-rater reliability was moderate to good for the UG (ICC 0.693 (95 % CI 0.580 to 0.788)) and good for the Dr G application (ICC 0.708 (95 % CI 0.597 to 0.799)).

**Conclusions:**

Moderate to high intra and inter-rater reliability of joint position and passive 1st MTPJ motion can be achieved with traditional and smartphone-based goniometric measurement. The Dr G application may provide a slightly higher reliability, but devices should not be used inter-changeably as significant variation in measurement between devices may occur.

**Electronic supplementary material:**

The online version of this article (doi:10.1186/s13047-015-0088-3) contains supplementary material, which is available to authorized users.

## Background

Satisfactory dorsiflexion at the first metatarsophalangeal joint (1st MTPJ) is essential to facilitate forward progression of the body during gait and to activate the windlass mechanism [1–3]. Measuring the angle of dorsiflexion available can also help identify the presence of pathology, such as hallux limitus or hallux rigidus [4]. Therefore, clinical assessment of the 1st MTPJ forms a fundamental part of a lower-limb biomechanical assessment [1, 5–7].

A variety of techniques can be used to assess 1st MTPJ range of motion including radiographic measurement, traditional goniometry, electromagnetic tracking and digitisation of video [8]. Reference values from radiographic studies for 1st MTPJ dorsiflexion of between 40° to 100° have been reported by Joseph [9] and 82° by Buell et al. [10]. However, these studies made no reference to the reliability of measurement. Although their study was very small (*n* = 6) Taranto et al. [8] report mean values of 1st MTPJ dorsiflexion up to 64° (SD 11) with high intra-rater reliability (r = 0.65 to 1.00) and high inter-rater reliability (r = 0.87). Several sources of error may affect the interpretation of radiographs including subject and beam position, selection of anatomical landmarks and construction of measurement lines [8, 11, 12]. Importantly, X-rays may not be routinely available to many clinicians in routine practice. Traditional hand-held goniometers remain the most commonly used tool in the clinical setting to assess joint position and range of motion [13–15]. These instruments are simple and quick to use and relatively inexpensive.

First MTPJ dorsiflexion angle is typically measured either with the clinician undertaking passive dorsiflexion of the hallux or the patient dorsiflexing their hallux actively while weight-bearing [16]. The dorsiflexion angle of the 1st MTPJ is typically established by using the medial midline of 1st metatarsal, proximal phalanx of hallux with 1st MTPJ as the fulcrum [6, 17]. A previous cadaveric study [18] reported a mean 1st MTPJ dorsiflexion of 76° which is in broad accordance with reference values for assisted dorsiflexion [10]. Previous work [19] indicated approximately 65° of dorsiflexion is required for normal gait. A value of less than 60° dorsiflexion suggests pathology of the 1st MTPJ using the grading system advocated by Coughlin and Shurnas [5]. However, lower values of 1st MTPJ dorsiflexion have been reported in-vivo. Hetherington and colleagues [20] recorded a mean value of 51° and Nawoczenski et al. [21] reported a value of 42°. The literature suggests the reliability of hand-held goniometric measurement can vary from moderate to good [16, 19, 22]. However, a range of factors can adversely affect the reliability of goniometric measurement, such as anatomical landmark identification, joint positioning and incorrect usage of the goniometer [21].

More recently smartphones have been developed with a sense acceleration and inclination in their software. This has enabled the development of clinical applications with goniometric properties, providing simple, faster measurement of joint position. The reliability of inclinometric measurements have been reported as similar, or superior to, that of traditional gonimetric measurements (for example, in the shoulder) [23–27]. This technology is increasingly being used within clinical practice, because it is quick, simple to read and may give the impression of superior accuracy [28–31]. Studies regarding the validity of digital measurements of joint deformity have reported similar or better results when compared to a traditional goniometer [32, 33]. However, in the foot the amount of current research into the reliability of smartphone applications is limited. The purpose of this study was to investigate the inter- rater and intra-rater-reliability together with the concurrent validity of a smartphone goniometric application compared with a traditional goniometer when measuring both joint position and passive motion of the 1st MTPJ.

## Methods

The Smartphone application used in this study was the Dr Goniometer (Dr G) application, (CDM S.r.L, Cagliari, Italy) together with a traditional, hand-held universal goniometer (UG). A double-blind, two- stage, repeated measures design was used. In stage one our aim was to determine if measurement of 1st MTPJ *position* by the two devices was reliable. In stage two we aimed to compare the reliability of both devices for assessing passive movement of the first MTPJ. Additionally, during both stages concurrent validity was assessed. The University of Brighton research governance and ethics panel approved the study and the GRRAS guidelines for reporting reliability [34] were followed.

### Participants

All participants were university students and were recruited through convenience sampling. Participants were included if they were in good general health and aged between 18 to 55 years. Exclusion criteria were: recent (past 6 months) lower limb injury, a structural disorder of the 1st MTPJ (e.g. hallux valgus), lower limb oedema, a history of degenerative or inflammatory joint disease, any neurological disorder, or recent foot surgery. Informed, written consent was gained from each participant. Participants only took part in one tier of this two-part study. A power calculation confirmed that a sample size of 25 subjects was required, which was broadly in line with previous work in this field [19].

### Raters

A total of eight raters were used in total. All raters were final year podiatry students and therefore had comparable levels of clinical experience. Importantly, all raters had received the same training in the use of goniometers. Prior to data collection a group training session on measurement of the 1st MTPJ was undertaken. This session appraised the validated methods of joint measurement [19, 35]; outlined the method used in this study and reviewed the manufacturers’ instructions for use of the UG and Dr G application.

### Measurement of 1st MTPJ position

**Fig. 1:**
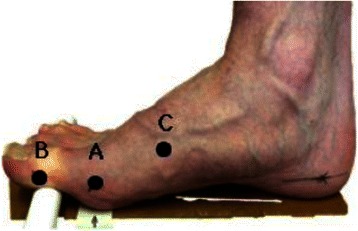
Location of skin markers

First MTPJ *position* was measured twice during a single session using both the hand-held goniometer and Dr G application by five raters (AB, KJ, SQ, SR, HW). Initially, relevant anatomical landmarks (medial aspect 1st MTPJ, inter-phalangeal joint and base of the 1st metatarsal) were palpated and marked by one researcher (RN) for each participant (Fig. 1). To ensure complete anonymity throughout, participants’ upper body remained shielded from view of all raters by the use of a privacy screen. One author (KH) positioned participants’ foot such that they stood on the same point on a raised platform (a handrail was available). This ensured each participant was in the same position prior to each measurement. The 1st MTPJ was then dorsiflexed by KH and a small prop placed under the hallux to maintain joint position throughout the measurement process.

Initially each rater measured the angle of dosiflexion using the UG. The angle display was in one- degree increments and was shielded, effectively blinding raters from the result. The central point of the UG was aligned over Point B and the end of the first arm was aligned with Point C (fixed arm). While holding the fixed arm securely in position, the second arm of the goniometer (moving arm) was aligned with Point A. Once satisfied with the positioning of the goniometer, it was handed to a single rater (WM) who read and noted the angle of dorsiflexion measured. Thus, raters were not aware of each other’s findings, removing the possibility of a Hawthorne effect.

**Fig. 2:**
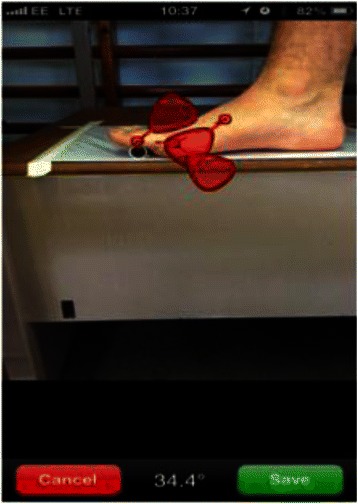
Screenshot of smartphone app

For the Dr G readings a smartphone (Apple iPhone 4 s, Apple Inc, Cupertino CA, USA) was cradled in an iPhone holder securely fixed to a raised stand, parallel to the floor and the same height as the platform participants stood on. The display was level with participants’ foot and its lens aligned with Point B (Fig. 1) at a distance of 30 cm. Each rater could place each of the three super-imposed markers of the Dr G application over points A, B and C on a picture taken by the smartphone, thus capturing an image of the joint position, (Fig. 2). Before each measurement the smartphone display was covered to blind raters from the result. Every measurement was read and noted by a single researcher (DW) who was also blinded to the measurements from the UG. The phone display was then cleared after each reading and was replaced in the same position for the next rater.

### Measurement of passive joint motion

To determine intra and inter rater reliability of passive joint *range of motion*, the same inclusion/exclusion criteria described above were employed and the same anatomical landmarks marked. As passive dorsiflexion of the 1st MTPJ was being assessed, subjects were seated on an examination couch, but foot positioning was ensured to be constant by one rater (RK). The full range of motion was measured as pilot work indicated it was not possible to consistently determine a standard start point (i.e. where the 1st MTPJ was neither dorsiflexed nor plantarflexed) to measure dorsiflexion alone. Therefore the 1st MTPJ was maximally plantarflexed prior to measurement. To ensure a standardised technique we adopted the approach advocated by Norkin and White [17] as detailed in Additional file 1. Three different raters (GH, NB, KW) measured passive 1st MTPJ range of motion twice during the same session using both the UG and Dr G. Again the scales for both devices were covered, effectively blinding raters from their own and each others measurements. As fewer raters were involved, measurements were taken in a random order, all measurements were noted and recorded by one person (RK). Prior to each measurement the UG was returned to its closed position and the smartphone display cleared by RK. Smartphone alignment and central position referencing was confirmed to be in the same position prior to each measurement by using the inbuilt smartphone inclinometer.

### Data analysis

Continuous data were entered into SPSS (v22, SPSS Inc, Chicago, Illinois) and checked for accuracy and normal distribution. Two entries from the smartphone data set for joint position were outliers and noted to be above what was considered anatomically possible (i.e. above 150°). These two data sets were discarded, as the risk of operator error could not be fully ruled out. No outliers were noted in the data set for joint range of motion. Descriptive statistics (mean, SD) were calculated for both devices for both maximially dorsiflexed 1st MTPJ position and range of motion. Pearson correlation coefficients were used to identify any significant associations between the two instruments when measuring 1st MTPJ motion. Intraclass correlation coefficients (ICCs) together with 95 % confidence intervals and the standard error of measurement (SEM) are reported to be best practice by Elliazew et al. [36]. The model of ICC used was ICC(2,k), where each subject was measured by each rater; and raters were considered representative of a larger population of similar raters [37]. The ICC values were interpreted accordingly: >0.75 indicates excellent; 0.4 to 0.75 indicates moderate-to-good reliability; and < 0.4 indicates poor reliability [38].

## Results

### Measurement of joint position

For the measurement of maximum 1st MTPJ dorsiflexion, a convenience sample of 26 healthy participants (15 male, 11 female, age range 18 to 51 years (mean age 27: SD 10.7)) was recruited. A total of 48 useable datasets were used, data from two participants were discarded as detailed previously and all data were normally distributed. The mean position of the 1st MTPJ measured with the UG was 32° (SD 8.02), whereas the mean measurement from the Dr G device was 27.8° (SD 11.7). Throughout the experiment the Dr G application consistently measured a smaller angle of 1st MTPJ dorsiflexion than the hand-held goniometer. Group-based data indicated that across the measurements the smartphone application consistently measured the joint angle on average 3.6° (SD 1.4) *lower* than the UG. The association in 1st MTPJ joint position measurements provided by these two instruments were significant (r = 0.399 *p* < 0.001). When assessing joint position, good intra-rater reliability (ICC >0.0.823) was noted for both devices and inter-rater reliability was excellent (ICC >0.785) for both devices (Table 1).

**Table 1:** Intra and inter-rater reliability for measuring *joint position*

	Universal goniometer	Smartphone app
1st MTPJ dorsiflexion Mean (SD)	Mean 32° (SD 8.37)	Mean 27.8° (SD 11.7)
Rater 1 Intra-rater reliability	ICC 0.861 (95 % CI 0.702 to 0.937) SEM 2	ICC 0.807 (95 % CI 0.603 to 0.912) SEM 1.5
Rater 2 Intra-rater reliability	ICC 0.709 (95 % CI 0.448 to 0.860) SEM 1.8	ICC 0.853 (95 % CI 0.689 to 0.934) SEM 1.5
Rater 3 Intra-rater reliability	ICC 0.829 (95 % CI 0.652 to 0.921) SEM 1.5	ICC 0.768 (95 % CI 0.537 to 0.893) SEM 2.0
Rater 4 Intra-rater reliability	ICC 0.857 (95 % CI 0.697 to 0.935) SEM 1.6	ICC 0.929 (95 % CI 0.836 to 0.969) SEM 1.6
Rater 5 Intra-rater reliability	ICC 0.861 (95 % CI 0.707 to 0.937) SEM 1.4	ICC 0.907 (95 % CI 0.796 to 0.959) SEM 1.3
Average inter-rater reliability	ICC 0.823 (95 % CI 0.642 to 0.918	ICC 0.853 (95 % CI 0.692 to 0.933)
Inter-rater reliability	ICC 0.785 (95 % CI 0.675 to 0.882)	ICC 0.832 (95 % CI 0.737 to 0.911)

*SEM* standard error of mean

*IC* inter class correlation coefficient

*CI* confidence intervals

### Measurement of passive joint motion

For measurement of passive dorsiflexion of the 1st MTPJ, a convenience sample of 32 healthy participants (15 males, 17 females, age range 18 to 51 years (mean 32; SD 10.1)) was recorded and no data sets were discarded. The mean range of motion at the 1st MTPJ measured by the two devices was almost identical (UG 82.8° (SD1 2.2): Dr G 82.9° (SD 11.3)). The average intra-rater reliability was excellent for both devices; the UG was ICC 0.809 and for Dr G application ICC 0.875. Regarding inter-rater reliability the mean for the UG was moderate to good (ICC 0.693), and excellent for the Dr G application (ICC 0.786) - Table 2.

**Table 2:** Intra and inter-rater reliability for measuring passive *joint motion*

1st MTPJ ROM	Universal Goniometer	Smartphone app
Mean (SD)	82.8° (12.2)	82.9° (11.3)
Rater 1 Intra-rater reliability	ICC 0.869 (95 % CI 0.793 to 0.918) SEM 1.6	ICC 0.869 (95 % CI 0.794 to 0.918) SEM 1.7
Rater 2 Intra-rater reliability	ICC 0.771 (95 % CI 0.650 to 0.854) SEM 1.6	ICC 0.886 (95 % CI 0.819 to 0.929) SEM 1.4
Rater 3 Intra-rater reliability	ICC 0.809 (95 % CI 0.672 to 0.864) SEM 1.4	ICC 0.870 (95 % CI 0.795 to 0.919) SEM 1.4
Average intra rater reliability	ICC 0.809 (95 % CI 0.705 to 0.879)	ICC 0.875 (95 % CI 0.803 to 0.922)
Inter-rater reliability	ICC 0.693 (95 % CI 0.580 to 0.788)	ICC 0.708 (95 % CI 0.597 to 0.799)

*ROM*, range of motion

*SEM*, standard error of mean

*ICC*, inter class correlation coefficient

*CI*, confidence intervals

## Discussion

To the best of our knowledge this study is the first to compare the reliability and concurrent validity of a smartphone goniometer application to a traditional goniometer when measuring dorsiflexion at the 1st MTPJ. Initially we aimed to determine if these devices were comparable when measuring joint position. When assessing static joint position reliability was good, concurring with the previous work on larger joints such as the shoulder [39], elbow [40] and knee [41, 42]; supporting the argument that the use of a robust protocol assists in reducing errors. Interestingly intra-rater reliability of the universal goniometer was slightly higher than that of the smartphone, but inter-rater reliability of the smartphone was higher than the goniometer. More importantly, the significant variation in results from these two instruments in relation joint position, suggests these devices should not be used inter-changeably. Similar findings were not replicated when assessing range of motion. However, the standard deviation and range of values were consistently greater for the UG, demonstrating increased variance in this set of results. All five testers had similar clinical experience and limited experience of using goniometers or smartphone-based applications in this context. The relatively low standard error of the mean across all raters (≤2°) suggests that this sample of measurement means is representative of the population of raters. That said, the Dr. G application does not require anatomical landmark identification, therefore clinicians may prefer to use this technology especially for those that are less experienced with measuring joint angles.

Inter-rater reliability is frequently lower than intra-rater reliability for clinical measurements often due to differences in technique [43, 44]. When measuring range of motion we found good intra-rater reliability for both devices and moderate to good inter reliability was recorded. The smartphone application demonstrated slightly greater reliability: however, the overlap of the 95 % confidence intervals associated with the two tool’s datasets prevents the assertion of any significant difference between the levels of reliability. We found reduced inter-rater reliability compared well with previous work [45], although the current study was in vivo, as opposed to using two-dimensional photographs. In a study of knee joint motion (a joint with predominantly flexion/extension motion) Jones et al. [44] found slightly greater improved reliability than in the current study. It may be that in larger joints the anatomy is easier to visualise and positioning of goniometers is more straightforward. Other potential causes of reduced reliability unique to using this type of technology include the recommended use of the inbuilt smartphone inclinometer whilst photographing the joint. Although the feature aims to align the lens with the joint axis of motion; alignment of the first metatarsal and phalanx on the ground and subsequent dorsiflexion of the joint is challenging and may cause the patient to engage muscles, which potentially restrict movement [46] and further work on reliability while not using the inclinometer is required.

This study is subject to some limitations. A major question is how much of the methods can be utilised in the clinical environment, as some elements may not be transferable due to factors of time and manpower. However, this work provides individual clinicians with a baseline protocol with known parameters of reliability and they may wish to develop and adapt this. It is important to note that 1st MTPJ anatomy is inherently variable. Additionally, we excluded structural deformity; and this may not fully reflect clinical practice. Owing to an experimental design being used to determine reliability between raters it was not possible to determine which device was most accurate. In terms of the transferability of this work, there may also be greater difficulty when assessing multi-planar joints. A single clinician may find it difficult to capture an image at the same time as testing range of motion [47]. It was beyond the scope of this study design to include participants with pathology or raters with varying levels of clinical experience. All raters in the current study had a similar level of clinical experience and it is unknown if more experienced clinicians would produce less variability. Although training and a pilot study was undertaken with both devices, it might be expected that there would be a period of learning by raters as they completed the task, regardless of level of experience. However, differences in the values between individual raters indicate that variations occurred throughout the experiment.

Pathology of the 1st MTPJ is often associated with a negative impact on individuals’ quality of life [48] and the reliability of goniometric measurements are fundamental if clinical decisions are based on these findings, particularly in the surgical context. Multidisciplinary teams increasingly deliver patient treatment and so measurement tools must have high levels of intra rater and inter rater reliability to have transferrable clinical significance. Elliazew and colleagues [36] have suggested that goniometric measurements only be considered valid if ICCs surpass 0.8 for inter-rater and 0.9 for intra rater reliability. Neither the inter-rater nor intra-rater reliability results in this study reached the standards of reliability proposed by Elliazew et al., for goniometric tools, although findings for the Dr G application were close to these levels. Further refinement of the protocols and additional training of assessors may yield greater levels of reliability.

## Conclusion

Using a detailed, robust protocol demonstrates that both hand-held and smartphone-based goniometric measurement of within session 1st MTPJ position and passive motion can be achieved with a moderate to high degree of intra and inter-rater reliability. It can be argued that smartphone applications such as Dr G, may provide a slightly higher degree of reliability. Importantly however, devices should not be used inter-changeably as significant variation in measurement between devices may occur.

## Additional file

Additional file 1:
**Protocol for the Dr G application and universal goniometer.**
