# Supplementary material for: The reliability of a smartphone goniometer application compared with a traditional goniometer for measuring first metatarsophalangeal joint dorsiflexion
Source: J Foot Ankle Res. 2015 Jul 23;8:30. doi: 10.1186/s13047-015-0088-3 (PMC4512018; doi:10.1186/s13047-015-0088-3)
Supplement: Additional file 1: — Protocol for the Dr G application and universal goniometer. [file 13047_2015_88_MOESM1_ESM.docx]

**Additional file 1**

Protocol for the Dr Goniometer app

1. The DG app was opened and the correct setting, (i.e. increments of measurement etc.) was selected by the rater who was reading all findings and the reading covered with tape.
2. The rater taking the measurement then placed the smartphone in the cradle & checked the distances.
3. The inbuilt inclinometer was aligned to parallel, which made sure the smartphone was always level before taking the photo.
4. The rater then dorsiflexed the 1st MTPJ to the end of it’s range of motion and took a photo.
5. The three markers of the Dr D app were then placed onto the photo, one along the medial aspect of the 1st ray, (point C); one of the medial aspect of the 1st MPJ (point B) and one along the medial aspect of the 1st toe (point A)
6. The angle created was then displayed at the bottom of the screen which was covered with opaque tape so that the rater could not see the measurement
7. The smartphone was handed to the rater who recorded the angle on the pre-prepared spreadsheet.

Protocol for using the plastic goniometer

1. The UG measurement scale was covered using a paper disc so that the rater could not see the measurement they have obtained
2. One arm of the UG was aligned along the medial aspect of the 1st ray (point C)
3. The centre of the pivot where the goniometer arms join was placed on the medial aspect of the 1st MTPJ joint (point B)
4. The second arm of the goniometer was aligned with the medial aspect of the 1st toe (point A)
5. The rater then dorsiflexed the 1st MTPJ to the end of its range of motion.
6. The UG was then passed to rater who read the measurement and recorded it on a pre-prepared spread sheet.
